# Accelerated radially encoded tissue phase mapping

**DOI:** 10.1186/1532-429X-16-S1-W21

**Published:** 2014-01-16

**Authors:** Jan Paul, Stefan Wundrak, Peter Bernhardt, Wolfgang Rottbauer, Volker Rasche

**Affiliations:** 1 Internal Medicine II, University Hospital of Ulm, Ulm, Germany

## Background

Velocity measurements of the heart muscle (Tissue Phase Mapping, TPM) can be used to quantify asynchrony or abnormal motion patterns [1]. However, long scan times restrict the clinical usability of these measurements. We investigate the use of accelerated radial acquisition to benefit from its unique motion artifact properties.

## Methods

In 3 healthy volunteers, a mid-level short axis slice was acquired at 3 T (Achieva, Philips) using a 32-channel cardiac coil. Two types of segmented, triggered, and gated velocity encoding sequences were used: a Cartesian acquisition (fully sampled; R = 1) and radial acquisitions with undersampling factors of R = 1,3, and 6. Acquisition parameters were: FOV = 340^2 ^mm^2^, resolution = 2^2^x8 mm^3^, acq. matrix = 172^2^, α = 15°, 3 k-lines/segment, gating window = 6 mm (except for the radial acquisitions in one volunteer: 8 mm), phase interval≈31 ms, 4-point balanced velocity encoding (Hadamard) with VENC = 30 cm/s. TR/TE = 6.3/4.6 ms (Cartesian) and TR/TE = 6.1/3.3 ms (radial). Reconstructed velocities were exported from the scanner. Higher undersampling was analyzed from a similar radial acquisition (R = 1) of a beating heart phantom [2] by leaving out profiles to simulate undersampling factors of R = 2 to 10. Each velocity encoding direction was reconstructed iteratively in Matlab by an intermediately regularized sparse SENSE algorithm with temporal total variation regularization [3]. Reconstructed in-plane and through-plane velocities were further processed in Matlab. Background phase errors were corrected according to a linear model. Median radial [v_r_(t)], circumferential [v_c_(t)] and longitudinal [v_l_(t)] velocities were obtained for each cardiac phase t and corrected by their mean velocity over time. Velocity curves were compared relative to fully sampled data (in vivo: Cartesian acquisition) by correlation coefficient CC. SNR of velocities within the myocardium was calculated as local mean to standard deviation ratio and averaged over all cardiac phases and volunteers.

## Results

**Figure 1:**
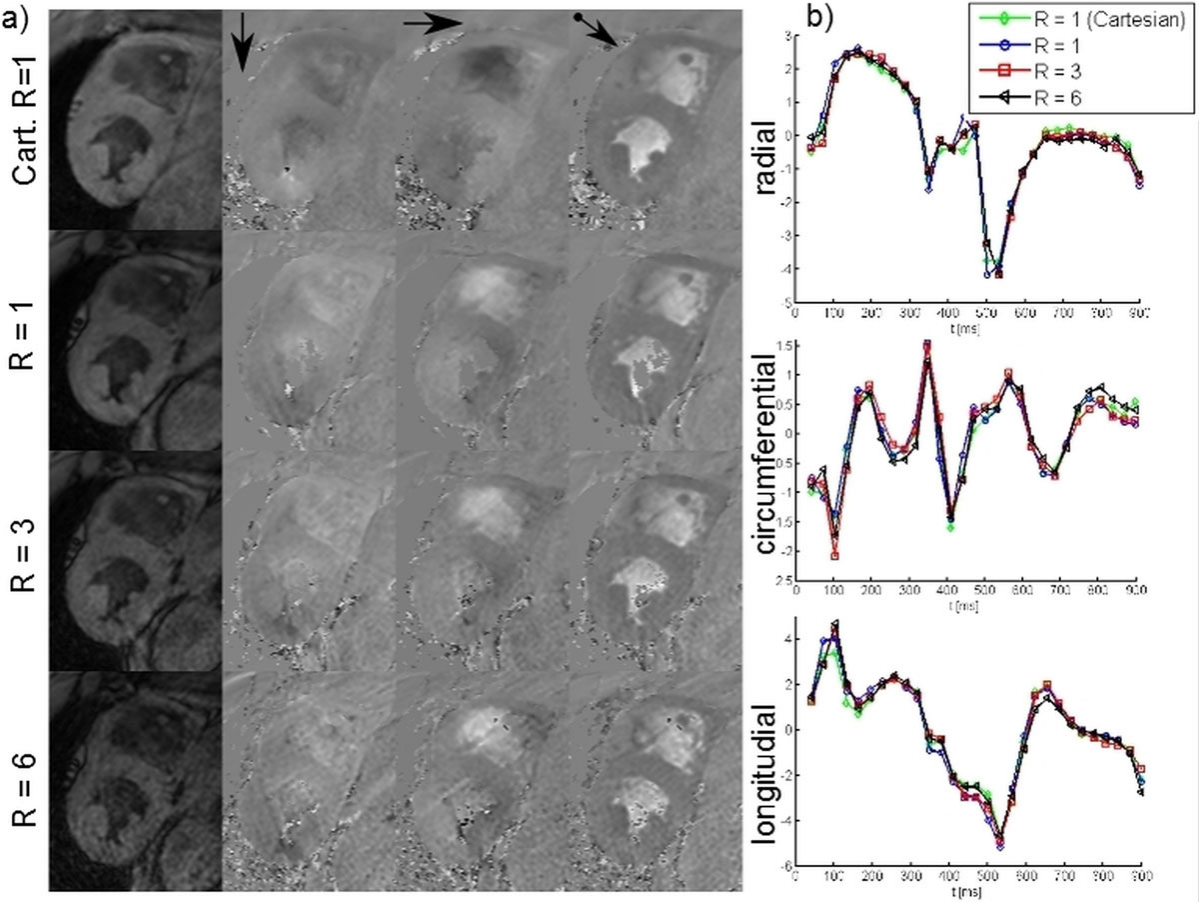
**(a) Anatomical and velocity images from Cartesian and radial acquisition in a volunteer**. Although noise is slightly increased for higher undersampling, overall image quality is preserved. (b) Median radial, circumferential and longitudinal velocities of the same volunteer show almost no difference.

**Figure 2:**
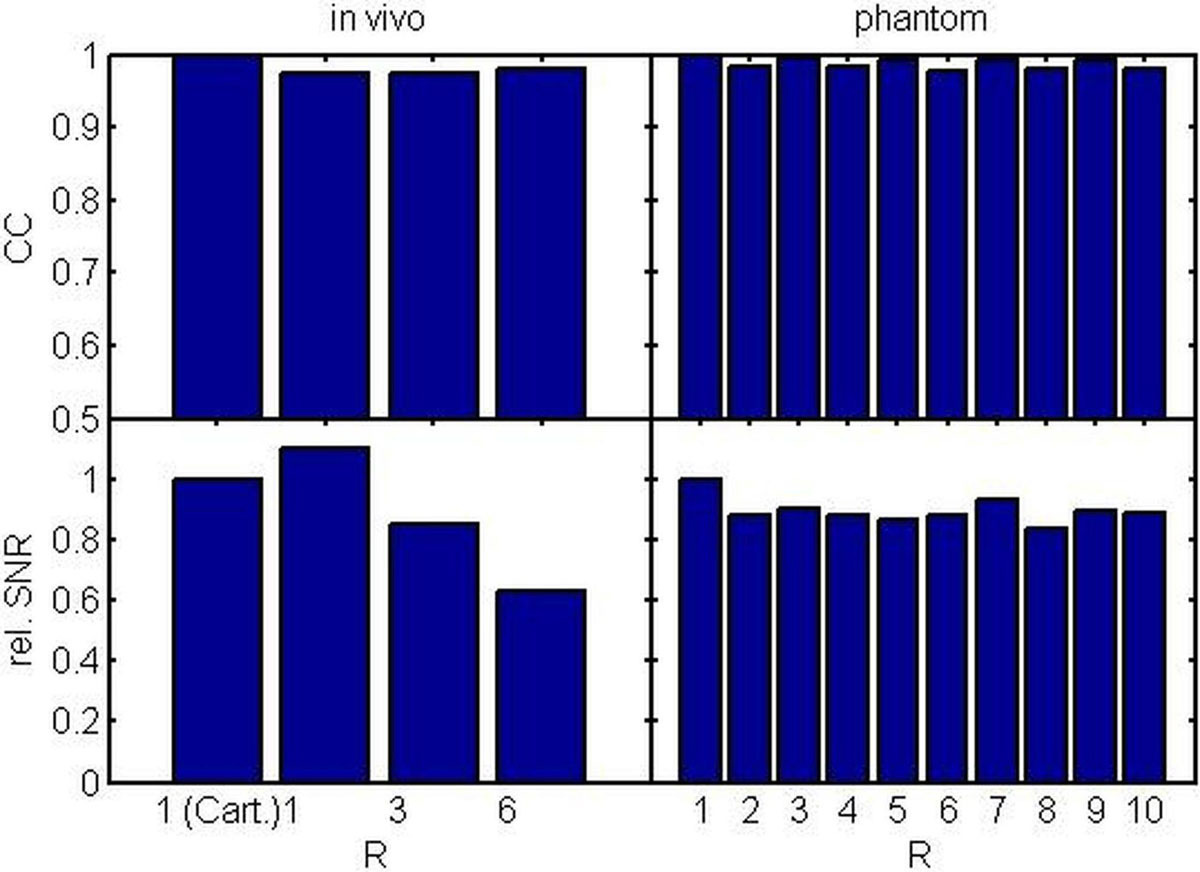
**Quantitative analysis of undersampling effects**. Top: Average correlation coefficient (CC) of median velocities with reference (fully sampled) shows no corruption of velocities in vivo and the phantom (all CC > .97). Bottom: Averaged signal to noise ratio (SNR) within the myocardium relative to fully sampled reference. While only small SNR loss is found in the simulated phantom undersampling (rel. SNR > .83), relative SNR in vivo is decreased to 63% for R = 6.

Figure 1a shows anatomical and velocity images exemplarily in one volunteer. Image quality is comparable for Cartesian and radial acquisition with all investigated undersampling factors, although noise is slightly increased for higher R. Median velocity curves (Figure 1b) give comparable results for all acquisitions. Quantitatively, SNR is decreased to 63% of the original value for in vivo data, but SNR loss for iteratively reconstructed phantom data is 17% at worst (see Figure 2). Median velocity curves are preserved (CC > .97, Figure 2) for all R in vivo and with the phantom.

## Conclusions

Radial TPM is feasible in vivo and yields comparable results to Cartesian TPM. Acquisition time can be saved by acquiring data with radial undersampling. Higher undersampling factors can be reached by using iterative reconstruction methods, as the phantom study suggests.

## References

[B1] Lutz JCMR 2012 14 74

[B2] Manzke ISMRM 2010 #3378

[B3] Wundrak ESMRMB 2012 #691

